# The role of N^6^-methyladenosine (m^6^A) mRNA modifications in herpesvirus infections

**DOI:** 10.1128/jvi.01723-24

**Published:** 2025-01-27

**Authors:** Ruth Verhamme, Herman W. Favoreel

**Affiliations:** 1 Department of Translational Physiology, Infectiology and Public Health, Faculty of Veterinary Medicine, Ghent University 366759 , Merelbeke, Belgium; New York University Department of Microbiology, New York, New York, USA

**Keywords:** herpes, m^6^A, epitranscriptome, virus-host interaction, viral replication, immune escape

## Abstract

Herpesviruses, a family of large enveloped DNA viruses, establish persistent infections in a wide range of hosts. This characteristic requires an intricate network of interactions with their hosts and host cells. In recent years, the interplay between herpesviruses and the epitranscriptome—chemical modifications in transcripts that may affect mRNA biology and fate—has emerged as a novel aspect of herpesvirus-host interactions. In particular, herpesviruses display different mechanisms to modulate and usurp the most abundant mRNA modification, N6-methyladenosine or m^6^A. Some herpesviruses interfere with m^6^A methylation of transcripts, while others enhance or take advantage of m^6^A methylation of viral and/or cellular transcripts. In many cases, herpesviruses appear to modulate the m^6^A methylation process to suppress the antiviral host response. This review highlights the strategies used by members of the different herpesvirus subfamilies to manipulate host m^6^A mediators and how these contribute to virus replication and the antiviral host response. Research aimed at deciphering the interaction of herpesviruses with the m^6^A epitranscriptome not only may lead to new avenues in the design of antiviral and immunomodulatory strategies, but also provides new insights in the regulation and the role of m^6^A transcript methylation in general.

## HERPESVIRUSES

Herpesviruses are large and complex viruses that contain a linear double-stranded DNA genome that is protected by an icosahedral capsid shell. This capsid is embedded in a tegument layer of viral and cellular proteins, which in turn is wrapped in a lipid envelope that is decorated with viral (glyco)proteins. On average, herpesvirus virions have a diameter of 150–250 nm (1, 2).

The *Herpesviridae* family contains more than 130 characterized viruses, divided into three subfamilies: *Alpha-*, *Beta-*, and *Gammaherpesvirinae*. Of these, alphaherpesviruses represent the largest subfamily and exhibit the fastest lytic replication cycle. This subfamily includes herpes simplex virus type 1 (HSV-1) and 2 (HSV-2) and varicella-zoster virus in man and several animal pathogens, such as the porcine pseudorabies virus (PRV) and the avian Marek’s disease virus (MDV). Betaherpesviruses have the most restricted host range and a slower replication cycle. Cytomegaloviruses, including human and mouse cytomegalovirus (HCMV and MCMV), belong to this subfamily (3). Gammaherpesviruses, like human Epstein-Barr virus (EBV) and Kaposi’s sarcoma-associated herpesvirus (KSHV), also have a relatively restricted host range. EBV is the first identified human oncovirus and contributes to approximately 2% of all human cancers (3, 4).

Herpesviruses share fundamental biological principles such as the structural features of their virus particles (virions), and all herpesvirus genomes contain a set of around 40 common genes. These core herpesvirus genes mostly encode structural components or proteins responsible for replication. Furthermore, all herpesviruses share the ability to undergo two distinct phases of infection, a lytic infection and a latent infection. Whereas the former results in the production of progeny virions, the latter results in a dormant infection, which allows herpesviruses to establish lifelong latent infections from which they may periodically reactivate. The three herpesvirus subfamilies are estimated to have arisen around 200 million years ago, around the time when mammals emerged from a line of mammal-like reptiles (5). As a result, herpesviruses have coevolved over millions of years with their hosts, which has resulted in highly complex and delicately balanced interactions with and manipulations of host cells and the host immune system (2).

## THE m^6^A EPITRANSCRIPTOME

Chemical modifications of DNA have been tracked down as soon as DNA was identified as the molecule carrying genetic information (6). These chemical DNA modifications were later shown to be critical for the regulation of protein synthesis, since epigenetic changes affect transcription efficiency. Over the past years, an additional regulatory layer of protein synthesis has been discovered, the epitranscriptome, which consists of chemical modifications of mRNA. Such RNA modifications were initially discovered in 1958, and over 150 different modifications have been identified in coding and noncoding RNAs by now (7, 8).

One of the best-known mRNA modifications is the 7-methylguanosine (m^7^G) cap, which consists of a methylation of the first guanosine nucleotide that shields the 5′ end of mRNA. This modification plays a crucial role in pre-mRNA processing, nuclear export, and cap-dependent protein synthesis by recruiting specific translation factors and protecting the mRNA from 5′ to 3′ exonuclease degradation (9). Furthermore, 2′-O-methylation of the nucleotide adjacent to the m^7^G cap serves as a “self” mark for the cellular mRNA to avoid recognition by the innate immune system (9, 10). Many viral transcripts also carry these modifications, complicating the ability of the cell to discriminate between self and viral mRNA (9, 10). Besides the cap modifications, it has become increasingly clear that viruses may also use and manipulate other mRNA epitranscriptome modifications to avoid or suppress cellular antiviral responses and/or to facilitate virus replication (11).

N^6^-adenosine methylation (m^6^A) is the most abundant internal modification of eukaryotic mRNA and is found in over 25% of transcripts in mammalian cells (12, 13). It is often enriched at the start of the last exon and is typically present in a conserved DR(m^6^)ACH consensus sequence (where D = A, G, or U, R = A or G, and H = A, C, or U) (12, 14, 15). In most cases, m^6^A is deposited onto mRNA by a multi-subunit complex called the m^6^A writer complex (12, 15). The catalytic core of the writer complex is composed of the actual m^6^A methyltransferase METTL3, the METTL14 protein that serves as an allosteric activator of METTL3, and the WTAP protein, a critical regulatory subunit that is responsible for the localization of the complex to nuclear speckles (16, 17). All components of the m^6^A writer complex are mainly located in the nucleus of the cell, where they can bind and directly methylate nascent mRNA (18). More recently, additional m^6^A RNA methylases have been identified: METTL16, which methylates MAT2A mRNA and U6 snRNA; METTL5, which methylates 18S rRNA; and ZCCHC4, which methylates 28S rRNA (19).

Some epitranscriptomic marks, including m^6^A methylations, can be removed by demethylases or so-called methylation eraser proteins. The fat mass and obesity-associated protein (FTO) was the first enzyme that was shown to demethylate m^6^A modifications on mRNA (20). Later, Mauer et al. showed that FTO displays a particularly high demethylase activity toward the N^6^,2′-O-dimethyladenosine (m^6^Am) modification that is found at the 5′-end of many mRNA and short nuclear RNA (snRNA) molecules (21). By that time, another m^6^A eraser protein was discovered, the ketoglutarate-dependent dioxygenase alkB homolog 5 (ALKBH5) (22). Similar to the m^6^A writer complex, eraser proteins like FTO and ALKBH5 are typically located in the nucleus.

Epitranscriptomic marks, like m^6^A, affect the biology and fate of transcripts since so-called reader proteins recognize and bind the specific epitranscriptomic mark. Binding of these reader proteins can influence splicing efficiency, transport, translation, and degradation rate of transcripts. The best characterized m^6^A reader proteins make part of the YTH domain-containing family of proteins, which contain a tryptophan cage that recognizes m^6^A (23–25). Of these, YTHDF1-3 is located in the cytoplasm, YTHDC1 is located in the nucleus, and YTHDC2 can be found in both the nucleus and cytoplasm of the cell. Although initial reports indicated that the YTHDF1-3 proteins bind different subsets of m^6^A-methylated transcripts and fulfill different functions, there are indications that these three types of YTHDF proteins act (partly) redundantly and mainly contribute to transcript degradation (26, 27). Beyond the YTH family, several additional proteins have been identified as (indirect) m^6^A readers, including the heterogeneous nuclear ribonucleoproteins (28) and insulin-like growth factor 2 mRNA-binding proteins (29).

**Fig 1:**
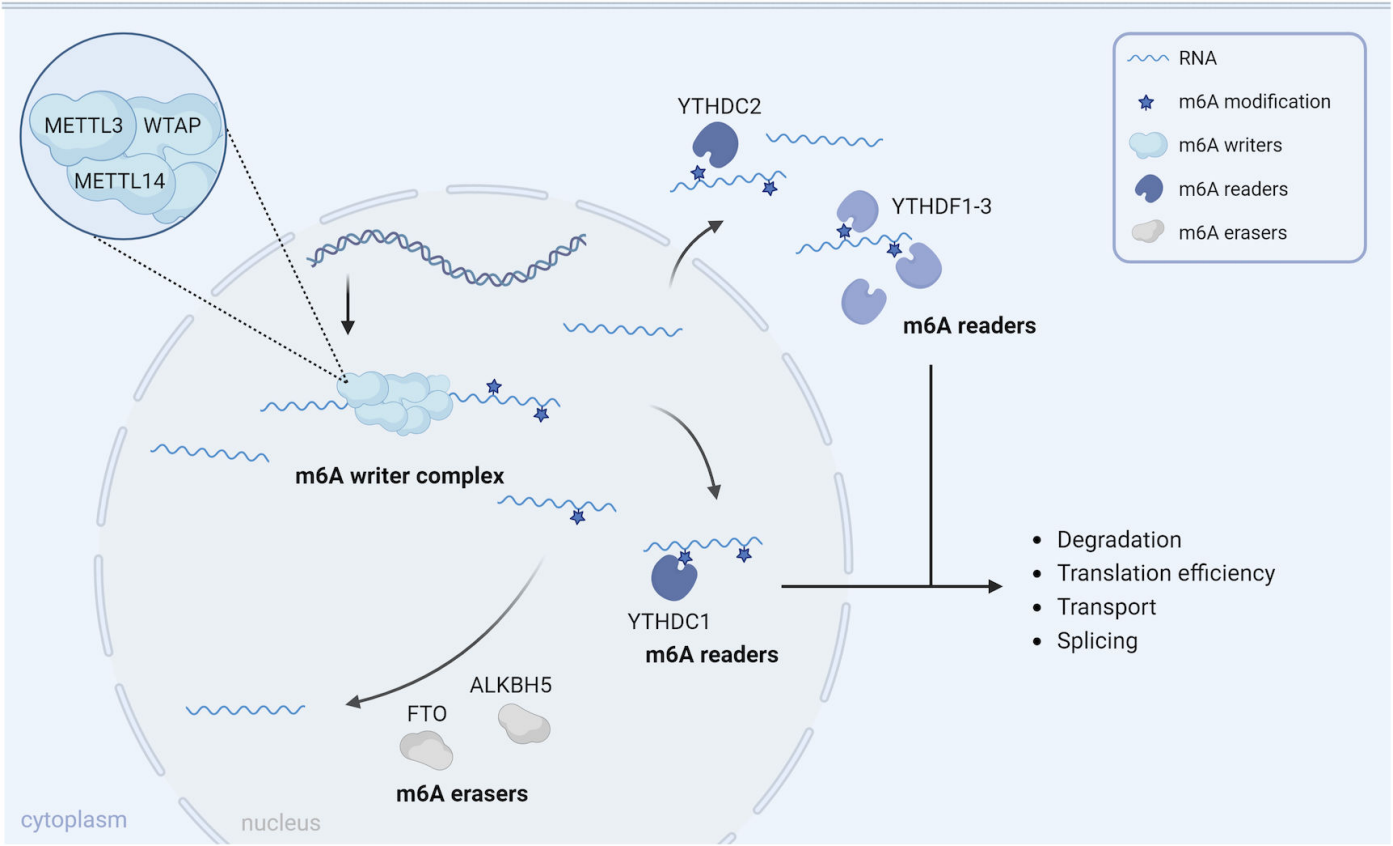
Overview of the cellular m^6^A machinery in uninfected host cells. The m^6^A writer complex, consisting of several subunits, among which the METTL3, METTL14, and WTAP core components, catalyzes the conversion of A to m^6^A on newly transcribed cellular mRNAs; The m^6^A eraser proteins FTO and ALKBH5 are capable of removing m^6^A; the m^6^A readers, such as YTHDF1-3 and YTHDC1-2, recognize m^6^A modifications on mRNA and influence the fate of the RNA, including RNA stability, transport, splicing, and/or translation. The figure was created using Biorender.com.

An overview of the cellular m^6^A machinery, including m^6^A writer, reader, and eraser proteins, is shown in Fig. 1.

## m^6^A METHYLATION OF HERPESVIRUS TRANSCRIPTS

The first reports of viral mRNAs that carry m^6^A modifications were obtained in the 1970s for influenza virus, adenovirus, and simian virus 40 (30–32). Given that m^6^A readers drive functional consequences of m^6^A-containing transcripts, m^6^A modification of viral transcripts might affect viral replication efficiency. It was rapidly assumed that viral m^6^A methylation was carried out by the cellular machinery since the consensus sequence that is methylated turned out to be the same for viral and cellular mRNA (33, 34). Herpesviruses, like most DNA viruses, replicate in the nucleus of the host cell, where they have access to the cellular m^6^A machinery.

### Alphaherpesviruses

In the 1970s, HSV-1 was the first herpesvirus that was shown to express m^6^A-methylated viral transcripts (35, 36). Interestingly, Bartkoski et al. discovered that late in HSV-1 infection, both viral and host cell transcripts no longer contain internal m^6^A methylations (35, 37). Jansens et al. recently found indications that there is an evolutionary pressure against the incorporation of the m^6^A methylation DRACH consensus sequence in the genomes of alphaherpesviruses, whereas betaherpesvirus and gammaherpesvirus genomes do not show evidence for such evolutionary pressure (38).

Despite the lack of information on the molecular mechanisms that drive m^6^A methylation at the time, the early reports in the 1970s already suggested that the lack of m^6^A methylation in transcripts in HSV-1-infected cells may be caused by a viral inhibition of the host enzyme(s) responsible for m^6^A methylation (35, 37). This hypothesis was confirmed recently, as different reports indeed showed that alphaherpesviruses impair the host m^6^A writer complex. Srinivas et al. reported that HSV-1 inhibits the m^6^A writer complex through subcellular relocation of several components of the m^6^A writer complex in infected fibroblasts (39). Chen et al. did not observe such m^6^A writer relocation but reported viral-induced ubiquitination and degradation of METTL14 in HSV-1-infected glioma cells (40). Jansens et al. showed that inactivation of the m^6^A writer complex in PRV- or HSV-1-infected epithelial cells depends on the viral serine/threonine protein kinase. Mechanistically, for PRV, US3 expression led to phosphorylation and thereby chromatin dissociation of key components of the m^6^A writer complex (38). US3-mediated phosphorylation and inactivation of the m^6^A writer complex was recently confirmed for the avian alphaherpesvirus MDV (41). In the spleen and thymus of MDV-infected chickens, lytic virus replication was associated with reduced expression of METTL3 and METTL14 (42). Somewhat contrary to the reports that point to alphaherpesvirus-induced inhibition/suppression of the m^6^A writer complex, Feng et al. showed that HSV-1 infection induced an upregulation of the m^6^A writer components METTL3 and METTL14 early in infection in HeLa cells, followed by a decreased expression later in infection, while expression of the m^6^A eraser proteins FTO and ALKBH5 were suppressed (43). In the latter study, siRNA-mediated depletion of m^6^A writer complex components suppressed virus replication, which was not observed in other studies (39, 44). Also, one study reported an increase in m6A methylation and observed m6A-methylated viral transcripts at 12 h post inoculation of epithelial cells with PRV, although these authors also noted a suppression of m6A methylation later in infection (45). The combined evidence suggests that although (early) viral transcripts may display m^6^A methylation, later stages of alphaherpesvirus infection are associated with inhibition of the m^6^A writer complex and concomitant low m^6^A levels in viral transcripts. In line with this, m^6^A methylation does not appear to be of paramount importance for alphaherpesvirus protein expression, at least in cell culture.

### Betaherpesviruses

Different studies found that infection with the betaherpesvirus HCMV is associated with an upregulation of the core subunits of the m^6^A writer complex in human fibroblasts (46, 47). In line with this, several HCMV transcripts carry m^6^A modifications. Specifically, 21 viral transcripts are m^6^A-methylated in wild-type fibroblast cells compared to METTL3 knockout cells (47). However, METTL3 depletion did not lead to significant changes in the expression of these viral transcripts (47), suggesting that m^6^A methylation of viral transcripts does not play a critical role in HCMV infection in cell culture. It is important to note that true METTL3 knockout cells are generally nonviable. Viable METTL3-depleted cell lines likely produce alternatively spliced functional METTL3 proteins that bypass the CRISPR-induced mutations (48).

### Gammaherpesviruses

A hallmark characteristic of all herpesviruses is their ability to establish latent infections, from which they may reactivate upon particular stimuli. During latency, the viral gene expression program is restricted, and no progeny virus particles are produced. For gammaherpesviruses, the role of m^6^A methylation has been particularly assessed in the context of reactivation of KSHV and EBV from latently infected cell culture models. Unfortunately, interpretation of some of these data is currently complicated by sometimes contradictory results, underscoring the need for additional studies to clarify some of the current uncertainties and ambiguities.

### KSHV

Multiple papers showed that viral transcripts of gammaherpesvirus KSHV are heavily m^6^A-methylated upon triggering lytic reactivation in latently infected cells and cell culture models by addition of doxycycline or 12-O-tetradecanoyl-phorbol-13-acetate (TPA), compared to viral transcripts in latently infected cells (49–53). Although, during KSHV reactivation in BCBL1 cells, there is no increase in METTL3 protein expression, a decreased FTO expression was observed, which could promote the preservation of m^6^A methylation marks in viral transcripts (49). BCBL1 cells are human B-cell lymphoma cells that carry KSHV latently and provide a widely used B-cell model of KSHV latency and reactivation. However, Tan et al. did not confirm the reduction in FTO expression upon lytic KSHV reactivation in closely related BCBL1-R cells—BCBL1 cells that express doxycycline-inducible replication and transcription activator (RTA) of KSHV—and showed stable expression of both the m^6^A writer and eraser proteins (53). Interestingly, shRNA-mediated knockdown of METTL3 or inhibition of general methylation by 3-deazaadenosine (DAA) in reactivated B cells interferes with the expression of viral transcripts, thereby leading to reduced viral protein expression and virion production (49). This suggests that m^6^A methylation of viral transcripts is critical for effective lytic KSHV infection in cell culture.

There is evidence to support a key role for RTA, the major viral transcriptional transactivator of KSHV, in this context. Indeed, RTA is likely involved in triggering m^6^A methylation during productive KSHV infection, since transfection of RTA in uninfected HEK293T cells results in a significant increase in overall m^6^A levels in transcripts, which is similar as seen during lytic KSHV infection in B cells (49). However, no indications were found for any type of interaction of RTA with the m^6^A writer complex or with m^6^A erasers as a putative explanation of how RTA could promote or preserve m^6^A methylation.

While RTA may be involved in m^6^A methylation of viral transcripts during KSHV lytic replication, m^6^A methylation is also important for efficient RTA expression. Functional inhibition of METTL3 during lytic KSHV reactivation results in reduced expression of viral proteins, including RTA. In line with the reported importance of m^6^A in mRNA splicing (49, 54), Ye et al. found that inhibition of m^6^A methylation by DAA treatment suppressed RTA mRNA but not pre-mRNA expression levels, indicating that the decrease in RTA expression upon inhibition of m^6^A methylation is caused by altered mRNA splicing (49). Furthermore, these authors showed that reactivation of lytic KSHV infection in the B-cell model leads to an interaction of YTHDC1 with m^6^A-methylated RTA transcripts. Since YTHDC1 recruits several splicing factors, such as SRSF3 and SRSF10, this interaction could lead to efficient splicing of RTA (49). Combined, these data suggest that m^6^A methylation is critical for effective mRNA splicing of RTA, which in turn is important for the further enhancement of m^6^A methylation that contributes to efficient lytic viral protein production in KSHV-infected B cells.

In line with this, Hesser et al. (51) observed that METTL3 knockdown resulted in reduced KSHV lytic mRNA and protein production and virus replication upon TPA-induced KSHV reactivation in iSLK.219 cells. These iSLK.219 cells are derived from SLK renal carcinoma cells, in which the KSHV transactivator RTA is expressed under the control of a doxycycline-inducible promoter, and which are latently infected with KSHV.219 virus that expresses GFP constitutively, whereas RFP is only expressed upon KSHV reactivation (55). The authors also noticed reduced RTA expression upon METTL3 knockdown (51). Furthermore, they showed that the knockdown of YTHDF2 also results in impaired viral transcript expression, including RTA transcripts, and reduced levels of infectious virions during the reactivation of KSHV infection. Interestingly, this was not the case for the other YTHDF reader proteins (51). In contrast, Tan et al. found that upon knockdown of YTHDF2, there is a fourfold increase of virion production in reactivated KiSLK cells (53) (like iSLK.293 cells, KiSLK cells are SLK cells that are latently infected with KSHV and express a doxycycline-inducible RTA transgene). The latter result was explained by an interaction of YTHDF2 with several m^6^A-methylated viral RNAs, as shown by RNA-binding protein immunoprecipitation assays. This interaction was proposed to lead to enhanced degradation of the transcripts, since siRNA-mediated knockdown of YTHDF2 resulted in an increased half-life of viral transcripts (53). These authors therefore propose that host cells might restrict KSHV lytic replication in cell culture by degradation of m^6^A-methylated viral transcripts via YTHDF2. Since similar approaches were used in both studies, the cause of these apparent contradictory results is currently unclear. Future identification of the m^6^A reader-viral transcript interactome in different infected cell models perhaps may shed light on which cellular and/or viral factors are involved in these apparent opposing roles for the m^6^A reader protein YTHDF2 in KSHV infection.

In addition to the well-established m^6^A reader proteins YTHDF1-3, YTHDC1, and YTHDC2, other proteins have been suggested to (indirectly) recognize and interact with m^6^A methylated transcripts. Interestingly, using a m^6^A-methylated hairpin in the KSHV RTA transcript, Baquero-Pérez et al. identified seven members of the Tudor domain-containing family of proteins as putative m^6^A reader proteins (50). Of these, Staphylococcal nuclease domain-containing protein 1 (SND1) showed the highest affinity for m^6^A-methylated RTA transcripts (50). Recent work revealed that m^6^A methylation of the viral RTA transcript leads to structural changes of the transcript, allowing it to interact with SND1 (56). Three spliceosomal proteins that are known to interact with SND1 showed increased interaction with the RTA transcript during lytic reactivation. However, SND1 was not involved in splicing of RTA, since knockdown of SND1 in B cell lymphoma cells did not lead to changes in spliced RTA transcripts (50). Nonetheless, upon shRNA-mediated knockdown of SND1 in doxycycline-reactivated BCBL1-R cells, unspliced m^6^A-methylated RTA transcripts were found to be more unstable, leading to reduced RTA protein and overall viral protein expression (50). This suggests that SND1 stabilizes the m^6^A-methylated KSHV RTA transcript, which is important for efficient lytic KSHV infection upon reactivation.

Of note, an m6A atlas has been described that provides an overview of m6A sites in different vertebrate species and several virus species, including KSHV (57).

### EBV

While some studies showed an overall downregulation of m^6^A RNA methylation and an associated downregulation of m^6^A writer and reader proteins in gastric carcinoma cells infected with the gammaherpesvirus EBV (58–60), other studies observed m^6^A methylation of EBV transcripts (61–63). Both lytic and latent transcripts are m^6^A-modified during either type of infection (61). Different stimuli can be used to trigger lytic EBV replication in latently infected cell cultures, including TPA which activates protein kinase C (PKC) and butyrate which inhibits histone deacetylases (HDACs). Interestingly, and quite contrary to the observations in KSHV, Lang et al. (61) showed a dramatic reduction in m^6^A-methylated transcripts upon TPA- and butyrate-triggered lytic EBV reactivation from latency in lymphoblastoid cell lines (LCLs, generated by EBV infection of B lymphocytes) and EBV-carrying Burkitt lymphoma Akata cells. Upon depletion of m^6^A methylation by shRNA-mediated knockdown of METTL14 or METTL3, an increase in expression of several lytic proteins and decrease of several latent proteins were observed in latently infected LCLs and Akata cells (61, 63).

In line with the notion that m^6^A methylation appears to promote latent EBV infection and suppresses lytic EBV infection, a recent study uncovered that SUMOylation of YTHDF2 correlates with enhanced degradation of lytic viral transcripts and that transfection of YTHDF2 and the ligase responsible for its SUMOylation leads to reduced viral DNA copies upon EBV reactivation in HEK293T cells (64). In addition to YTHDF2, also YTHDF1 and YTHDF3 are SUMOylated upon lytic EBV reactivation (64). Knockdown of YTHDF1 via siRNA or reduction of SUMOylation of YTHDF1 or YTHDF3 by mutation of the SUMOylation sites during EBV reactivation in CNE2EBV cells (EBV-carrying poorly differentiated nasopharyngeal carcinoma cells) and Akata cells results in increased virus production (58, 64). Furthermore, knockdown of YTHDF1 increased the half-life of EBV transcripts, and YTHDF1 interacts with different proteins involved in the decapping of transcripts in lytic reactivated CNE2EBV cells (58). Therefore, Xiao et al. (58) suggest that YTHDF1 destabilizes lytic viral mRNAs, possibly by decapping, thereby suppressing EBV lytic replication.

Zheng et al. showed that m^6^A methylation of the transcript encoding EBV nuclear antigen 2 (EBNA2), a viral protein expressed during particular types of EBV latency, is needed for its efficient expression in latently infected B-cell lymphoma Raji cells, since shRNA knockdown of METTL3 led to a decrease in expression of the EBNA2 protein, while knockdown of the m^6^A eraser FTO led to an increase in EBNA2 expression (63). In line with this, EBNA2 transcripts interact with the YTHDF1-3 reader proteins in pull-down assays in latently infected B cell lymphoma cells (63). Knockdown of YTHDF2 via shRNA in latently infected B-cell lymphoma cells results in decreased EBNA2 transcript and protein levels, while knockdown of YTHDF1 and 3 results in their increased expression (63). This suggests that m^6^A reader proteins might regulate the stability of this viral transcript, although the exact influence of each m^6^A reader protein appears to be different, despite the reported overlap in YTHDF protein functionality (27).

Overall, these data indicate that m^6^A methylation of viral transcripts plays a role in the initiation and/or maintenance of EBV latency and that depletion of m^6^A methylation enhances reactivation, at least in cell culture.

However, Yanagi et al. (65) reported somewhat contrasting results since, in their assays, knockout of METTL3 in latently infected Akata cells did not lead to changes in the expression of the latency protein EBNA1, nor of any lytic protein. As mentioned higher, results using METTL3 knockout cells should be interpreted with caution since viable METTL3 knockout cell lines are thought to produce alternatively spliced functional METTL3 proteins that circumvent the CRISPR-induced mutations (48). Nonetheless, upon triggering reactivation of latent EBV by anti-IgG treatment, METTL3 knockout resulted in a decrease of latent and lytic protein expression and reduced virion production compared to reactivation in wild-type Akata cells. Furthermore, these authors found that general inhibition of methylation via DAA treatment also results in decreased viral protein expression in reactivated Akata cells. Of interest, upon triggering of EBV reactivation from Akata cells, a significant increase in cleaved caspase-3 was seen in METTL3 KO cells compared to wild-type Akata cells, suggesting that METTL3-mediated m^6^A methylation contributes to suppression of apoptotic cell death during lytic EBV infection (65). Hence, although m^6^A methylation of viral transcripts may promote EBV latency in cell culture, m^6^A methylation may also play a role in (some cell culture models of) EBV reactivation by suppressing apoptotic cell death.

In line with an important role for m^6^A methylation during EBV latency in cell culture, different studies show higher levels of m^6^A writer complex subunits during latent EBV infection compared to cells in which reactivation is triggered (61, 66). Lang et al. (61) found that in LCL and Burkitt lymphoma cells that are latently infected with EBV, there is an increase in METTL14 expression compared to uninfected cells and to EBV-infected cells that undergo reactivation. In Akata and HEK293T cells, a subtle increase in METTL14 expression was observed during latent EBV infection. Reduced METTL14 expression upon lytic reactivation correlates with the dramatic reduction in m^6^A-methylated transcripts upon reactivation from latency seen in LCL and Akata cells. The viral latency protein EBNA3C may be involved in METTL14 upregulation and stabilization in latently infected cells since EBNA3C transfection in Saos-2 cells increased METTL14 mRNA and protein levels. In addition, EBNA3C interacts with METTL14 in BJAB cells that stably express EBNA3C and in EBV-transformed LCL cells (61). The circular EBV RNA circRPMS1, which is mainly expressed during latency, may also contribute to efficient m^6^A methylation during EBV latency, since Zhang et al. (67) showed that transfection of circRPMS1 triggers increased METTL3 expression and thereby increases m^6^A levels in gastric carcinoma (GC) cells. Mechanistically, circRPMS1 contributes to enhanced m^6^A methylation by recruiting a transcriptional regulator to the METTL3 promotor, inducing its expression (67).

In apparent contrast to some of the other studies, Xiao et al. and Xu et al. observed an overall decrease of m^6^A levels in total RNA in EBV-infected GC cells (58, 59). Xiao et al. (58) showed that latent EBV infection suppresses WTAP expression in GC cells compared to non-infected GC cells, correlating with reduced m^6^A levels. Transfection of the viral small RNA EBV encoded RNA 1 (EBER1) was sufficient to reduce WTAP expression levels (58). However, Xu et al. did not observe changes in WTAP expression but showed increased FTO levels (59). The differences observed in these studies compared to the other reports likely can be attributed to differences in infected cell types and latency programs.

## IMPACT OF HERPESVIRUS INFECTION ON THE HOST m^6^A EPITRANSCRIPTOME

m^6^A mapping studies have indicated that highly stable cellular household transcripts typically lack m^6^A, whereas dynamic transcripts, which are rapidly but temporarily expressed upon specific environmental cues, are often m^6^A-methylated, pointing toward an important role for m^6^A in regulating rapid and temporal gene expression (68). Therefore, it may come as no surprise that several antiviral immune-related transcripts are m^6^A-methylated, since these are rapidly and temporarily expressed upon virus recognition. As such, m^6^A methylation may help to ensure a tight regulation of the antiviral response (47, 69), while viruses may manipulate the host m^6^A epitranscriptome to restrain this response.

### Alphaherpesviruses

The first line of the immune defense against herpesviruses involves the recognition of herpesviral DNA by pattern recognition receptors that activate host antiviral pathways. The sensor proteins IFI16, cGAS, and hnRNPA2B1 recognize viral DNA and activate the antiviral type I interferon (IFN) response and the inflammasome (70–73). Interestingly, hnRNPA2B1 not only serves as a viral DNA sensor but may also function as an m^6^A reader protein that traffics m^6^A-methylated cGAS, IFI16, and STING mRNAs from the nucleus to the cytoplasm upon HSV-1 infection, thereby further promoting the type I IFN response (73). It is conceivable that this m6A-dependent role of hnRNPA2B1 is not restricted to HSV-1 but likely holds true for many, if not all, DNA viruses.

Alphaherpesvirus infection has a prominent impact on the host m^6^A epitranscriptome. While Wang et al. observed increased m^6^A levels due to METTL3 upregulation upon HSV-1 infection (74) and Feng et al. observed both hyper- and hypomethylation of transcripts in HSV-1-infected cells (75), Jansens et al. showed that infection of epithelial cell lines with either HSV-1 or PRV leads to a significant loss of m^6^A levels in total mRNA (38). The latter study confirmed early research by Bartkoski and Roizman who discovered that late in HSV-1 infection, neither viral nor host transcripts contain internal m^6^A methylation marks (35, 37). In addition, by mapping the host transcriptomes of infected versus uninfected cells to known host transcript m^6^A methylation patterns, infection of cells with either HSV-1 or PRV was found to result in preferential depletion of m^6^A-methylated host transcripts (76). This selective depletion of host m^6^A-methylated transcripts depends on the cytoplasmic YTHDF m^6^A reader proteins and is believed to occur via YTHDF-mediated degradation of m^6^A-containing transcripts in cytoplasmic mRNA processing bodies (P-bodies) (76). Since several antiviral IFN-related host transcripts are m^6^A-methylated, and since YTHDF depletion results in strongly increased expression of IFN and IFN-stimulated genes (ISGs) (47, 69, 76), it is thought that preferential depletion of m^6^A-containing host transcripts during PRV and HSV-1 infection constitutes a viral immune evasion mechanism (76). In line with this hypothesis, knockdown of YTHDF2 in HSV-1-infected cells results in enhanced ISG expression and thereby decreased viral protein production (77).

Further in line with alphaherpesvirus-mediated and m^6^A-dependent suppression of IFN and ISG expression, depletion of the m^6^A eraser protein ALKBH5 by siRNA was linked with increased IFN and ISG expression as well as increased STING signaling in HSV-1-infected cells and consequently reduced HSV-1 replication (78). Late in HSV-1 infection, ALKBH5 protein expression levels were reduced (78), which the authors suggested to serve as a mechanism of the host to interfere with the ongoing viral replication. Li et al. reported post-translational modification, specifically lactylation of ALKBH5, and showed that lactylated ALKBH5 binds IFN-β transcripts during HSV-1 infection, thereby leading to IFN-β demethylation and enhanced mRNA stability (79). Recently, it was shown that PRV infection has an impact on the FTO m^6^A(m) demethylase, which again correlates with reduced antiviral ISG expression. More specifically, expression of the viral UL13 serine/threonine protein kinase of PRV is required and sufficient to trigger phosphorylation of FTO, which correlates with a UL13- and FTO-dependent suppression of ISG expression in PRV-infected primary epithelial cells (80).

**Fig 2:**
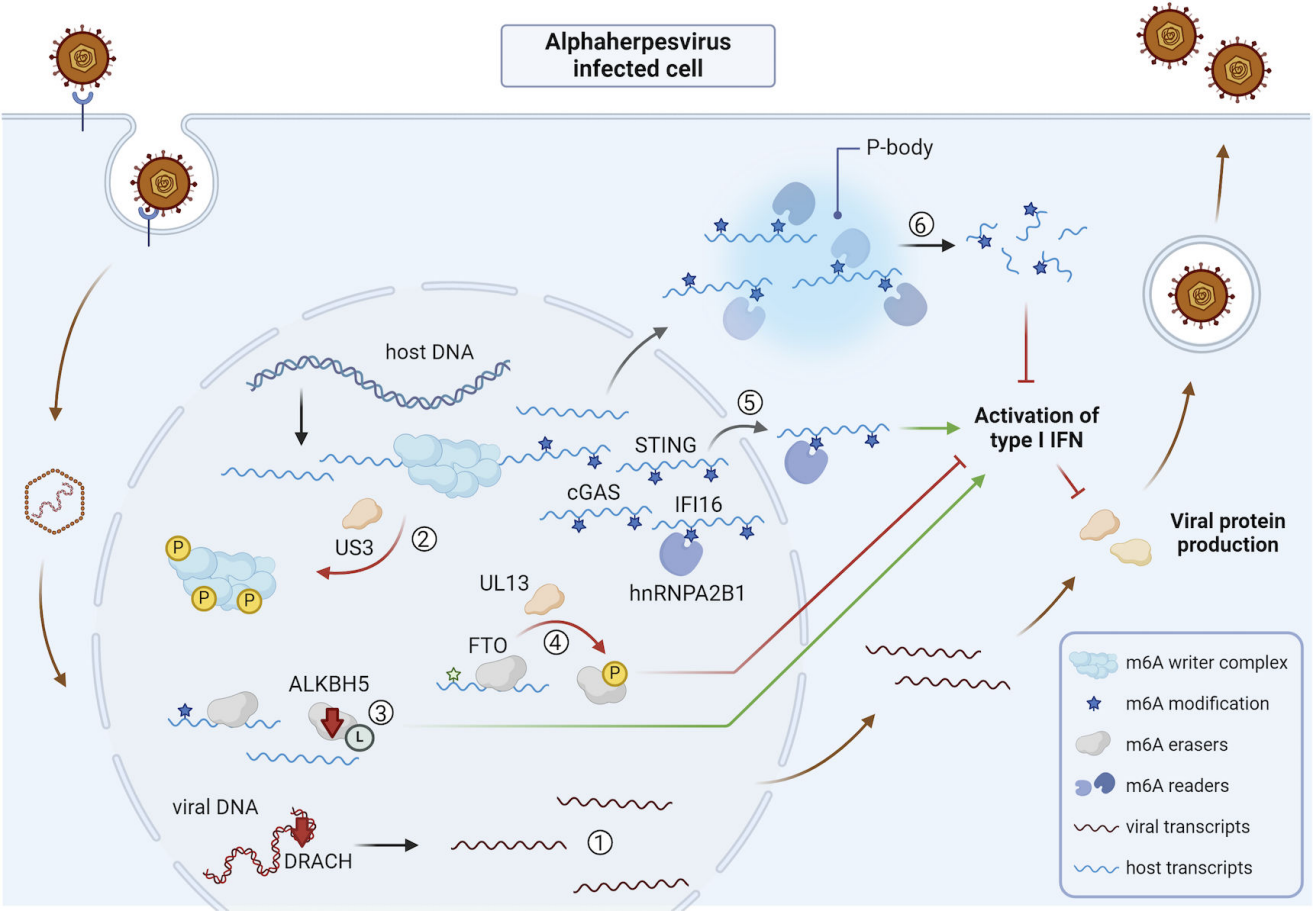
Overview of the impact of alphaherpesvirus infection on the viral and host m^6^A epitranscriptome and m^6^A machinery. ① Late in HSV-1 and PRV infection, viral (and host) transcripts do not contain m^6^A modifications (35, 37, 38), and there is an evolutionary pressure against the incorporation of the DRACH consensus sequence in the genomes of alphaherpesviruses (38). ② Infection with HSV-1, PRV, or MDV leads to inactivation of the m^6^A writer complex, contributing to the absence of m^6^A-methylated transcripts late in infection. The conserved alphaherpesvirus kinase US3 triggers phosphorylation and chromatin dissociation of core subunits of the complex in PRV-infected cells, correlating with its inactivation (38). In HSV-1-infected cells, relocalization of several subunits of the m^6^A writer complex was observed (39). ③ Reduced protein expression levels of the m^6^A eraser ALKBH5 late in HSV-1 infection (78) and lactylation of ALKBH5 during HSV-1 infection (79) are linked with an increased IFN response. ④ In PRV-infected cells, the m^6^A eraser protein FTO is phosphorylated via the viral protein kinase UL13, which correlates with suppression of ISG expression (80). ⑤ hnRNPA2B1 serves as a host m^6^A reader protein and traffics m^6^A-methylated cGAS, IFI16, and STING transcripts, which are involved in the antiviral immune response, to the cytoplasm during HSV-1 infection. This transport contributes to the activation of the type I IFN response (73). ⑥ HSV-1 and PRV infection both lead to preferential depletion of m^6^A-methylated host transcripts via the YTHDF1-3 proteins that relocalize to enlarged P-bodies. This is linked to reduced ISG expression (76). Abbreviations: HSV-1, herpes simplex virus 1; PRV, pseudorabies virus; ALKBH5, alkB homolog 5; cGAS, cyclic GMP-AMP synthase; STING, stimulator of interferon genes; IFI16, interferon-gamma inducible protein 16; P-body, RNA processing body; L, lactylation. The figure was created using Biorender.com.

Together, these data suggest that alphaherpesvirus infection affects m^6^A reader and eraser proteins to selectively downregulate m^6^A-methylated host transcripts and suppress the antiviral host IFN/ISG response. An overview of the impact of alphaherpesvirus infection on the viral and host m^6^A epitranscriptome and m^6^A machinery is shown in Fig. 2.

### Betaherpesviruses

As mentioned before, HCMV infection of human fibroblasts leads to increased levels of m^6^A writer complex components (46, 47). Although expression of HCMV transcripts that carry m^6^A methylation marks is not substantially affected upon depletion of METTL3, knockout or knockdown of METTL3, METTL14, or WTAP leads to decreased HCMV virus production (46, 47). In addition to m^6^A writer complex components, HCMV infection is also associated with increased levels of the YTHDF1-3 and YTHDC1 reader proteins in human fibroblasts (46, 47). In line with the effects of m^6^A writer knockdown/knockout, separate knockout of each of the YTHDF reader proteins leads to reduced virus production, further supporting the notion that an infection-associated increase in m^6^A mediators may contribute to efficient HCMV replication (47). Combined, these data suggest that m^6^A methylation of host transcripts, rather than viral transcripts, may be of particular importance in HCMV infection.

Rubio et al. (46) and Winkler et al. (47) showed that IFN-β transcripts that are produced shortly after HCMV infection of fibroblasts are m^6^A-methylated. This was observed as an m^6^A peak in IFN-β sequences upon immunoprecipitation of m^6^A-methylated RNA fragments (MeRIP-seq, methylated RNA immunoprecipitation sequencing). In METTL3 knockout cells, this m^6^A peak in IFN-β transcripts was reduced, leading to increased transcript stability and IFN-β protein production at 24hpi compared to that in parental cells (47), which highlighted the important regulatory role for m6A methylation in the antiviral IFN response in general. As a consequence, METTL14 knockdown or METTL3 knockout led to enhanced IFN-β mRNA expression and increased ISG expression, which correlated with decreased HCMV virion production in infected fibroblast cells (46, 47). Interestingly, inhibition of IFN signaling by the addition of a Janus kinase inhibitor rescued virus production in METTL3- or METTL14-depleted fibroblast cells (46, 47). Together, these results show that HCMV appears to benefit from the m^6^A writer complex, as m^6^A methylation lowers IFN-β transcript stability and protein production, leading to reduced ISG induction and therefore more efficient virion production (46, 47).

Increased m6A methylation in HCMV-infected cells may possibly have negative consequences. Like in fibroblasts, infection of vascular endothelial cells with HCMV results in increased levels of METTL3 and m^6^A methylation. This was associated with increased m^6^A methylation of transcripts encoding ubiquitin carboxyl-terminal hydrolase-L1 (UCHL1) and mitochondrial calcium uniporter (MCU). In the case of UCHL1 transcripts, this was associated with mRNA decay and reduced protein levels (81), whereas MCU m6A methylation was associated with increased protein levels (82). Both effects appear to contribute to HCMV-induced endothelial cell damage (81, 82).

Curiously, HCMV infection is also associated with increased levels of the m^6^A eraser proteins FTO and ALKBH5 late in infection in human fibroblasts (46). Knockout of each of the eraser proteins leads to increased viral titers whereas knockout of the writer subunits leads to reduced virus production (47). The upregulation of the m^6^A eraser proteins therefore does not appear to be favorable for the virus and may possibly serve as a host mechanism to counteract the virus-induced upregulated m^6^A writer complex expression.

**Fig 3:**
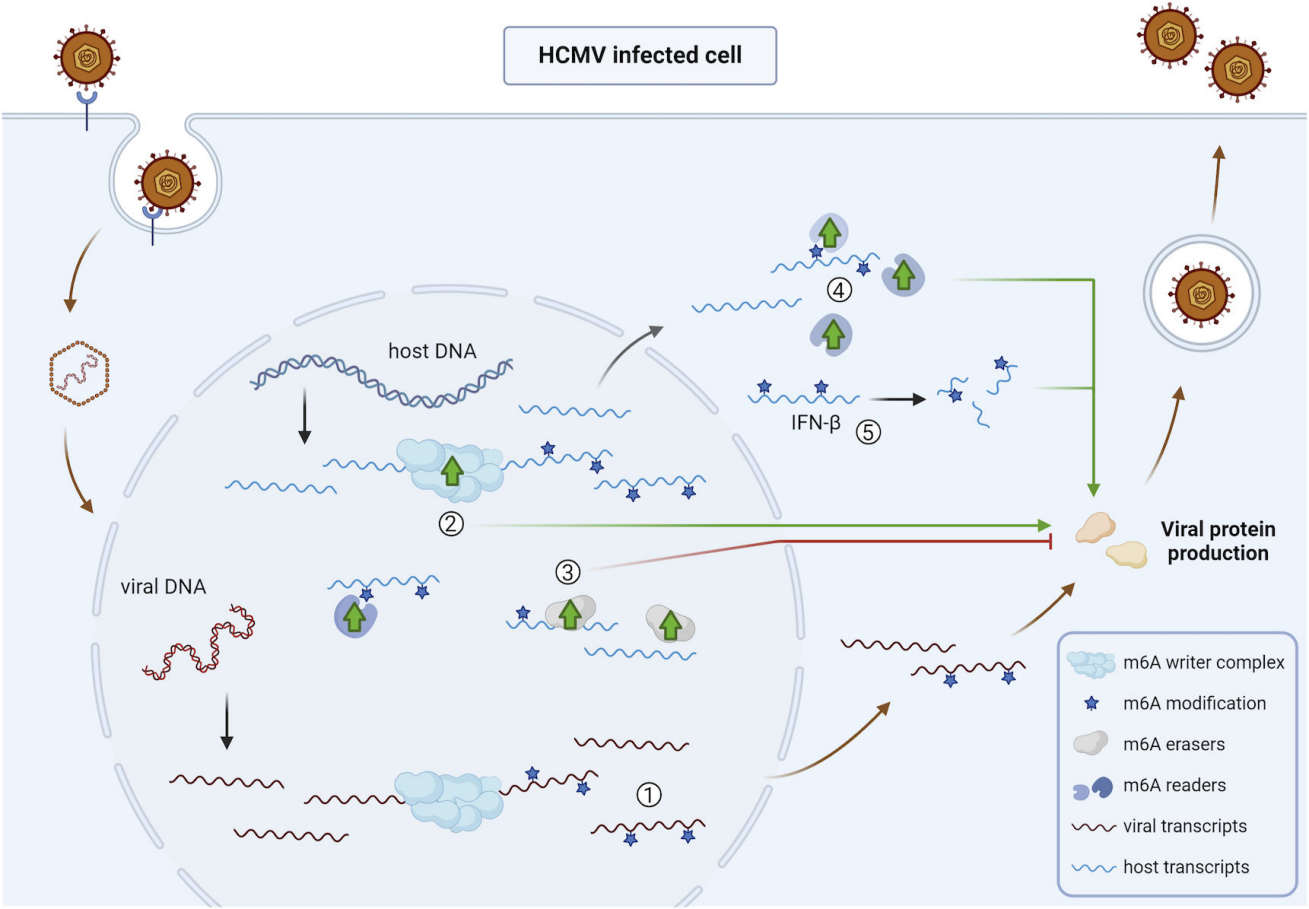
Overview of the impact of HCMV infection on the viral and host m^6^A epitranscriptome and m^6^A machinery. ① Several viral transcripts of HCMV are m^6^A-methylated (47). ② Infection of HCMV leads to an upregulation of the core subunits of the m^6^A writer complex, which is linked with increased virus production (46, 47). ③ HCMV infection triggers an upregulation of the m^6^A erasers FTO and ALKBH5 late in infection, which is linked with decreased virus production (46, 47). ④ YTHDF1-3 and YTHDC1 are upregulated during HCMV infection, possibly linked to increased virus production (47). ⑤ The cellular IFN-β transcripts that are produced shortly after HCMV infection are m^6^A-methylated, leading to reduced mRNA stability and protein production and thereby contributing to more efficient virus replication (47). Abbreviations: HCMV, human cytomegalovirus; IFN-β, interferon beta. The figure was created using Biorender.com.

An overview of the impact of HCMV infection on the viral and host m^6^A epitranscriptome and m^6^A machinery is shown in Fig. 3.

### Gammaherpesviruses

Gammaherpesvirus infection also alters the host m^6^A epitranscriptome, as redistribution of m^6^A modifications on host transcripts was reported during both KSHV and EBV infection (51, 62, 63).

### KSHV

Doxycycline-induced lytic reactivation of KSHV in latently infected iSLK.219 cells resulted in a substantial decrease of m^6^A deposition on cellular transcripts (51). Other studies confirmed the reduction in host m^6^A levels upon KSHV reactivation in this cell line as well as in BCBL1-R cells (52, 53). Since most studies so far have focused on the role of m^6^A methylation in viral transcripts, little is known about the mechanisms or consequences of this reduction in m^6^A levels in host transcripts during KSHV reactivation.

Although the general host m^6^A profile is suppressed, some *de novo* m^6^A-methylated host transcripts were detected in KiSLK and BCBL1-R cells 48 h after induction of lytic reactivation (53). In line with this, Macveigh-Fierro et al. (52) showed via MeRIP-seq that upon lytic reactivation of SLK.219 cells by doxycycline, the host interleukin-6 (IL-6) transcript gains m^6^A deposition at the 3′ UTR. This additional m^6^A modification is located in the so-called SOX resistance element that is present in the IL-6 transcript (52). SOX is a KSHV endoribonuclease that is conserved throughout the gammaherpesvirus subfamily and is involved in mRNA degradation to interfere with host gene expression (83). The m^6^A modification in the SOX resistance element promotes IL-6 escape from degradation by SOX, suggesting that this mechanism serves as a host countermeasure to interfere with the viral host shutoff during KSHV infection (52).

Manners et al. (84) showed, again via MeRIP-seq, an increased m^6^A peak in the cellular G protein-coupled receptor class C group member A (GPRC5A) transcript upon doxycycline-induced KSHV reactivation in BCBL1-R cells, which correlated with increased GPRC5A mRNA and protein expression. In line with this, shRNA-mediated knockdown of FTO led to increased GPRC5A mRNA abundance, while knockdown of WTAP or YTHDF1 resulted in decreased mRNA abundance. Furthermore, transfection of a plasmid encoding GPRC5A mutated in the four DRACH sites of the differentially modified m^6^A peaks led to reduced mRNA and protein levels compared to wild-type GPRC5A transfection in HEK293T cells. GPRC5A is a member of the G-protein-coupled receptors and has a role in inhibition of the pro-inflammatory nuclear factor kappa B (NFkB) signaling pathway, suggesting that KSHV-induced m^6^A modification of GPRC5A transcripts and the associated increased mRNA and protein levels may serve as a viral strategy to suppress the NFkB signaling pathway (84).

Li et al. (79) reported that, similar to the alphaherpesvirus HSV-1, KSHV infection triggers lactylation of ALKBH5, and showed that lactylated ALKBH5 binds IFN-β transcripts during infection, thereby leading to IFN-β transcript demethylation and enhanced mRNA production (79). Accordingly, this post-translation modification of the m^6^A eraser enhances the type I IFN response during KSHV infection. Since ALKBH5 lactylation was observed not only in HSV-1- and KSHV-infected cells, but also in mpox-infected cells (79), this may point to a more general host cell response to curb virus replication by increasing the innate IFN response.

Interestingly, one study explored the role of METTL16, an RNA methyltransferase responsible for m^6^A methylation of host MAT2A mRNA, during KSHV infection. MAT2A is a rate-limiting enzyme in the methionine-S-adenosylmethionine (SAM) cycle that is required for transmethylation of protein, DNA, and RNA. METTL16-mediated m^6^A methylation of MAT2A transcripts is essential for its expression. Knockdown of METTL16 (or of MAT2A) promoted KSHV lytic replication in KiSLK cells, possibly through increasing intracellular reactive oxygen species levels, which could be reversed by SAM treatment (85). These results therefore point to a suppressive role for METTL16 in KSHV lytic replication. Further studies are warranted to confirm the role of METTL16 in KSHV infection and to investigate whether similar mechanisms operate in other herpesviruses.

**Fig 4:**
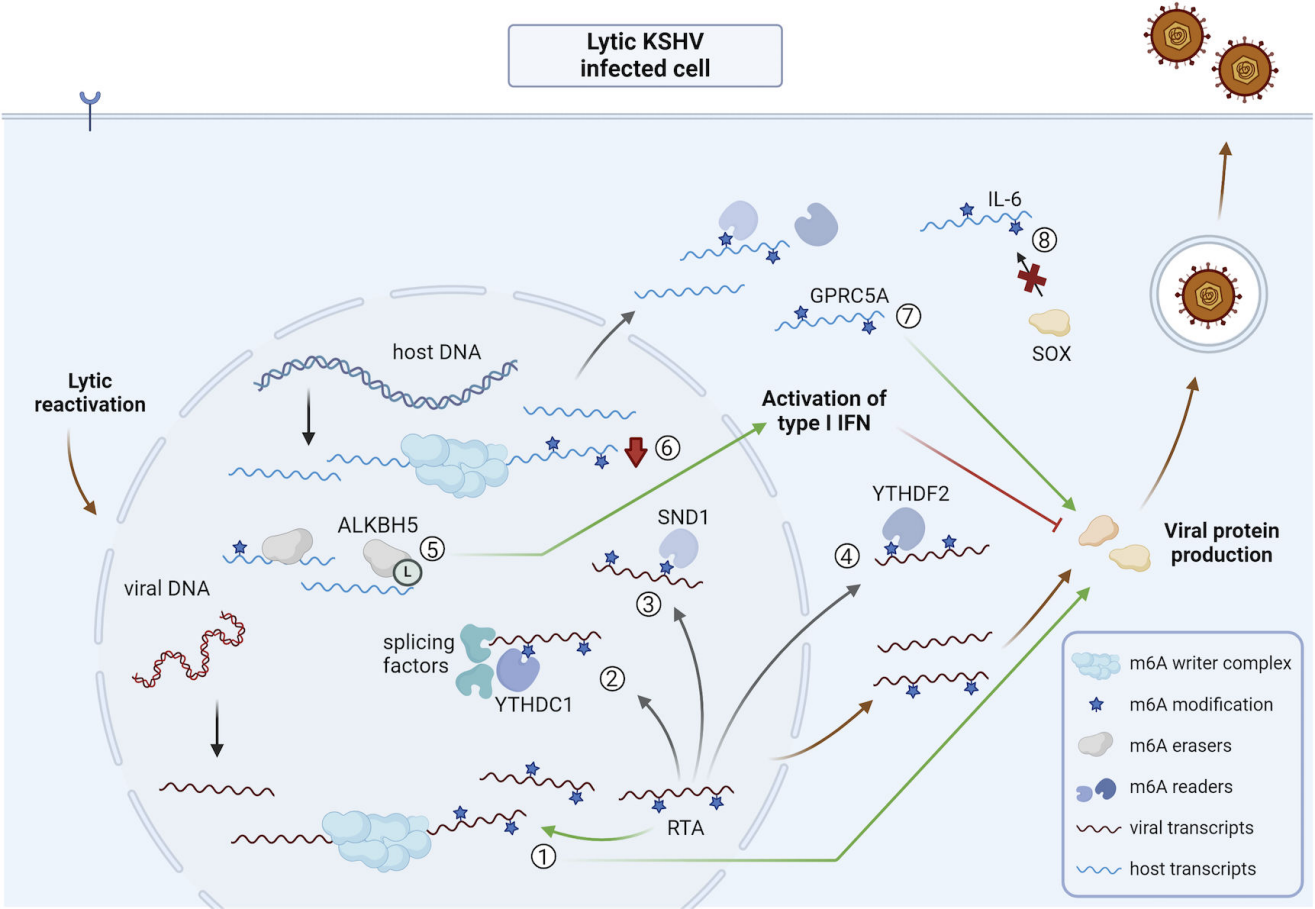
Overview of the impact of lytic reactivation of KSHV on the viral and host m^6^A epitranscriptome and m^6^A machinery. ① Viral transcripts of KSHV are heavily m^6^A-methylated upon reactivation from latency, which is linked to enhanced viral protein expression (49–53). One of the m^6^A-methylated transcripts is the major viral transcriptional transactivator RTA, which is involved in the increase in m^6^A methylation during infection, possibly contributing to efficient viral protein production (49). ② m^6^A-methylated RTA interacts with YTHDC1. Since YTHDC1 interacts with splicing factors such as SRSF3 and SRSF10, and m^6^A methylation of RTA influences its splicing, this interaction could lead to effective RTA splicing (49). ③ The m^6^A reader SND1 interacts with m^6^A-methylated RTA leading to increased stability of the pre-spliced transcript (50). ④ YTHDF2 interacts with m^6^A-methylated viral transcripts, although the impact of this interaction is not fully understood (51, 53). ⑤ KSHV infection triggers lactylation of ALKBH5. Lactylated ALKBH5 interacts with IFN-β transcripts, leading to IFN-β demethylation and enhanced mRNA production (79). ⑥ KSHV reactivation leads to reduced m^6^A methylation of many cellular transcripts (51–53). ⑦ GPRC5 transcripts gain m^6^A deposition leading to increased expression which is linked with more efficient viral replication (84). ⑧ IL-6 transcripts gain m^6^A deposition at the SOX resistance elements rendering these transcripts protected from degradation by the viral exonuclease SOX (52). Abbreviations: KSHV, Kaposi’s sarcoma-associated herpesvirus; RTA, replication and transcription activator; SND1, Staphylococcal nuclease domain-containing protein 1; IL-6, interleukin-6; GPRC5A, G protein-coupled receptor class C group 5 member A; L, lactylation. The figure was created using Biorender.com.

An overview of the impact of lytic KSHV infection on the viral and host m^6^A epitranscriptome and m^6^A machinery is shown in Fig. 4.

### EBV

Dai et al. (86) found reduced METTL3 mRNA levels upon lytic EBV infection in nasopharyngeal epithelial cells. The EBV immediate early protein BZLF1 is thought to be involved in this suppression of METTL3 expression, since METTL3 promotor activity is reduced upon transfection of BZLF1 in HEK293T cells. In addition, a recent study showed that, via the viral protein EBNA2, EBV triggers the proteasomal degradation of METTL3 in infected BJAB cells (87). As a consequence, EBV infection leads to reduced m^6^A methylation of several cellular transcripts. Indeed, EBV infection of BJAB cells resulted in a significant downregulation of 2,586 m^6^A peaks in the cellular transcriptome as assessed by MeRIP-seq (whereas 918 m^6^A peaks were significantly upregulated) (63).

One of the transcripts that loses m^6^A methylation during EBV infection encodes Toll-like receptor 9 (TLR9), a critical pathogen recognition receptor involved in viral DNA recognition. Although reduced m^6^A methylation typically leads to increased transcript stability, in this particular case, loss of m^6^A methylation was associated with reduced TLR9 transcript stability, which in turn correlates with reduced TLR9 protein expression in EBV-infected primary B cells (63, 87). Reduced m^6^A methylation was also observed in transcripts encoding the cellular transcription factor Krüppel-like factor 4 (KLF4) during lytic EBV infection of nasopharyngeal epithelial cells. In this case, reduced m^6^A methylation had a more canonical effect as it led to increased KLF4 expression (86). KLF4 is a negative regulator of the host antiviral response as it inhibits IFN-β and ISG expression (88). In line with this, overexpression of KLF4 leads to increased spread of EBV, suggesting that reduced m^6^A methylation of the KLF4 transcript and consequent increased protein expression are beneficial for viral spread during lytic EBV infection (86). Although the underlying mechanisms, the specificity, and overall consequences of EBV-induced depletion of m^6^A methylation from individual host transcripts are still unclear, these data suggest that at least some of these effects suppress the host antiviral response.

As mentioned, in addition to the downregulation of many m^6^A peaks in the host epitranscriptome, Zheng et al. also showed a significant upregulation of 918 m^6^A peaks in the cellular transcriptome upon lytic EBV infection of BJAB cells (63). One of these transcripts encodes FAS, a protein involved in apoptosis induction. Increased m^6^A methylation of FAS transcripts was associated with increased transcript stability in infected BJAB cells which in turn correlated with increased protein expression in primary B cells upon EBV infection (63). Further in line with an upregulation of m^6^A methylation of specific transcripts upon lytic EBV replication, Bose et al. (89) showed that lytic EBV reactivation from latency by IgG treatment in LCLs and Akata cells leads to enhanced m^6^A methylation of deltex E3 ubiquitin ligase 4 (DTX4) and tyrosine kinase 2 (TYK2) transcripts, encoding two key players involved in the IFN signaling pathway. For DTX4, a suppressive factor of IFN, enhanced m^6^A methylation was linked with enhanced protein expression. In contrast, increased m^6^A methylation of transcripts encoding TYK2, a Janus kinase that contributes to IFN signaling, was associated with downregulation of the corresponding protein (89). Interestingly, YTHDF2 shRNA knockdown rescued TYK2 protein expression in reactivated LCL and Akata cells, suggesting that EBV reactivation triggers enhanced m^6^A methylation and subsequent YTHDF2-mediated degradation of TYK2 transcripts. Along the same lines, a recent study showed that lytic EBV infection triggers YTHDF3-induced degradation of the m^6^A-methylated ISG transcript IFN-induced transmembrane protein 1 (IFITM1) through DDX5 (90). Since IFITM1 inhibits EBV entry, suppressed expression of IFITM1 leads to increased viral infection (90). Yang et al. (90) showed that the role of IFITM1 in EBV infection is specific to epithelial cells, highlighting that differences in experimental outcomes regarding the impact of virus infection on the host epitranscriptome and its consequences for virus replication may to some extent be related to differences in cell types.

**Fig 5:**
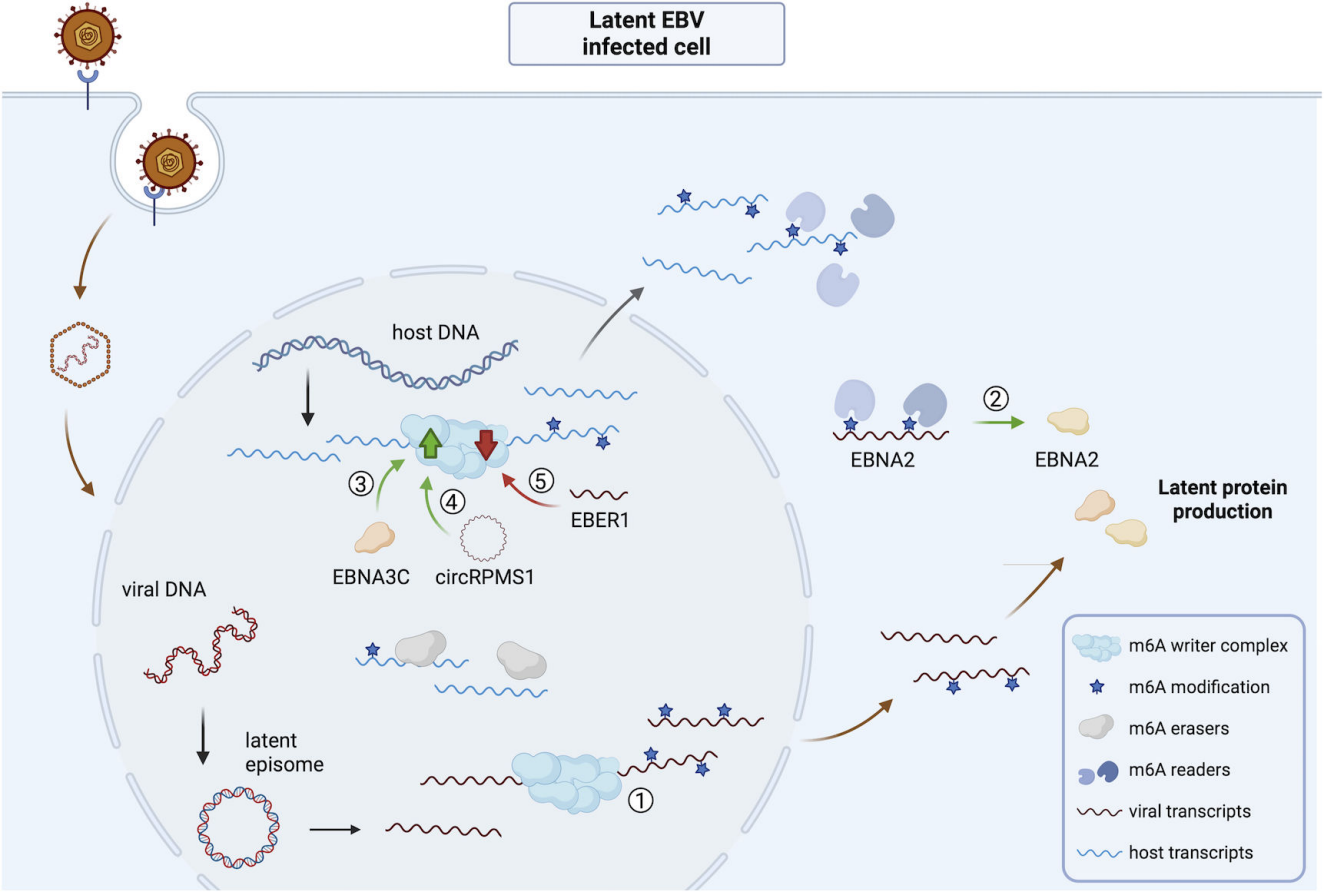
Overview of the impact of latent EBV infection on the viral and host m^6^A epitranscriptome and m^6^A machinery. ① Several viral transcripts of EBV are m^6^A-methylated during latent infection (61, 62). ② The latency-associated and m^6^A-methylated EBNA2 transcript interacts with YTHDF1-3, and m^6^A methylation correlates with enhanced EBNA2 protein expression (63). ③ The latent EBNA3C protein binds METTL14 and triggers its upregulation, correlating with efficient latent protein production (61). ④ The circular RNA circRPMS1 triggers upregulation of METTL3 and thereby m^6^A levels, which is linked to efficient latent protein expression (63, 67). Hence, the m^6^A writer complex seems to be important for efficient maintenance of EBV latency in cell culture. ⑤ In contrast, EBER1, a viral small noncoding RNA, triggers reduced protein expression of WTAP (58). Abbreviations: EBV, Epstein-Barr virus; EBNA3C, EBV nuclear antigen 3C; EBER1, EBV encoded RNA 1; EBNA2, EBV nuclear antigen 2. The figure was created using Biorender.com.

**Fig 6:**
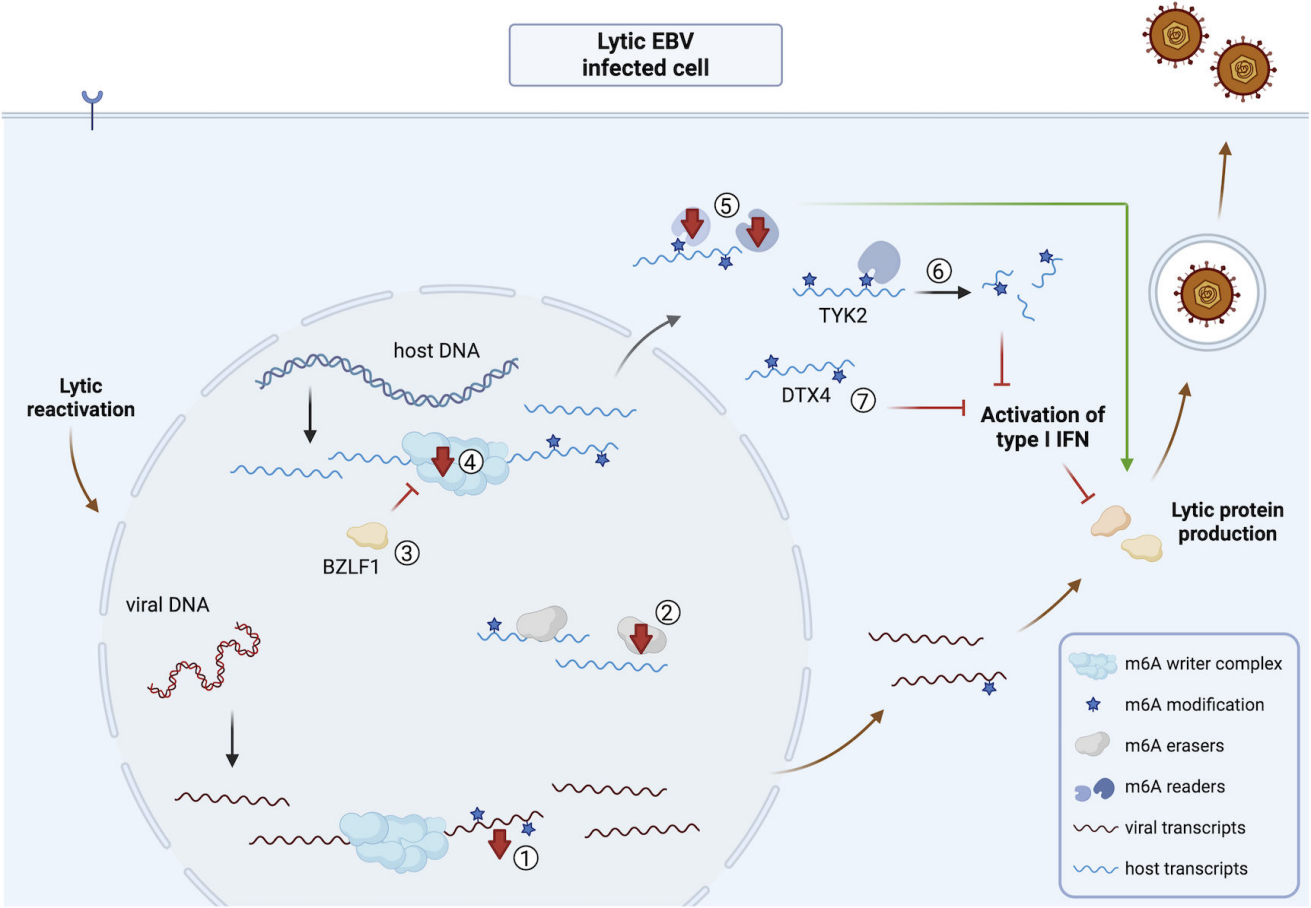
Overview of the impact of lytic reactivation of EBV infection on the viral and host m^6^A epitranscriptome and m^6^A machinery. ① There is a strong reduction of m^6^A-methylated viral transcripts upon EBV reactivation from latent to lytic infection, although some viral transcripts still contain m^6^A methylations during lytic infection (61, 62). ② Reactivation triggers downregulation of ALKBH5 (61, 89). ③ The immediate early protein BZLF1 inhibits METTL3, leading to reduced m^6^A levels and thereby influencing host transcript stability (86). ④ Reactivation triggers a reduction in METTL3 and METTL14 protein levels, correlating with reduced m^6^A levels without affecting viral protein production during lytic EBV infection (61, 66). ⑤ YTHDF1-2 are downregulated, correlating with enhanced virus production (62, 64, 66, 86). ⑥ A key activating factor in IFN signaling is TYK2, which also shows enhanced m^6^A methylation upon reactivation. This is associated with enhanced YTHDF2-mediated degradation of TYK2 transcripts, thereby suppressing the antiviral IFN response (89). ⑦ DTX4, a key suppressive factor in the IFN signaling pathway, shows enhanced m^6^A methylation, which correlates with enhanced protein expression which in turn is linked to a suppressed IFN response (89). Abbreviations: EBV, Epstein-Barr virus; DTX4, deltex E3 ubiquitin ligase 4; TYK2, tyrosine kinase 2. The figure was created using Biorender.com.

An overview of the impact of EBV infection on the viral and host m^6^A epitranscriptome and m^6^A machinery is shown in Fig. 5 for latent EBV infection and Fig. 6 for lytic EBV infection.

It is clear that the impact of gammaherpesvirus infection on the host m^6^A epitranscriptome is complex and appears to be finetuned, where both EBV and KSHV infection leads to reduced overall m^6^A levels in the host epitranscriptome but where both viruses also trigger increased m^6^A methylation of specific host transcripts. Although there are indications that the combination of both phenomena contributes to optimal virus replication/reactivation and immune evasion, it will be crucial to decipher the underlying molecular mechanisms to fully understand the role of these modulations in m^6^A methylation of host transcripts in virus biology and pathogenesis.

## CONCLUSIONS AND PERSPECTIVES

The field of viral interactions with the m^6^A epitranscriptome, and the entire field of epitranscriptomics, is recent and rapidly expanding. In addition, some of the generated data are somewhat contradictory, likely because of differences in *in vitro* models and other methodologies. Interpretation of (sometimes conflicting) results is further complicated by progressive insights in the epitranscriptomic field and by the natural growing pains associated with the development of novel methodologies to mine a recently developing field. For example, whereas m6A reader proteins YTHDF1, YTHDF2, and YTHDF3 were initially thought to display separate functions, there are indications that the functional palette of the YTHDF proteins may be (largely) overlapping (26, 27). The use of different m^6^A detection methods may be an important source of variance in the results, as different assays exhibit a trade-off between precision and recall. Different assays depend on m6A-targeting antibodies, which may suffer from insufficient specificity and typically also recognize m6Am methylation. Several review papers provide overviews and comparisons of different methodologies in epitranscriptomic research (91–93). Variations in sensitivity, specificity, and resolution between these techniques may influence the interpretation of m6A modifications and their biological impact. Despite these challenges, some general contours of the impact of herpesvirus infection on m^6^A methylation of transcripts and the role of m^6^A methylation in herpesvirus infections are starting to emerge, where different herpesviruses manipulate m^6^A methylation to suppress the antiviral IFN response and where differences in m^6^A methylation patterns contribute to the switch in the latency/reactivation viral protein expression program in human gammaherpesviruses.

Although an increasing number of reports describe the complexities and importance of the interaction of herpesviruses with m^6^A methylation of mRNA, it has to be pointed out that these reports currently focus on only a few members of this very large virus family. Indeed, reports are largely limited to HSV-1, PRV, and MDV for the alphaherpesviruses, HCMV for betaherpesviruses, and EBV and KSHV for gammaherpesviruses. It will therefore be important to include additional members of different species within these subfamilies, and possibly also members of the more distantly related *Alloherpesviridae* (herpesviruses of fish and amphibians) and *Malacoherpesviridae* (herpesviruses of bivalves) families of the *Herpesvirales* order, to be able to discern common, similar, and distinct interactions of these highly successful pathogens with the m^6^A epitranscriptome.

Another important element to point out is that a defining characteristic of all herpesviruses is their ability to establish lifelong latent infections in their natural host, from which they may reactivate upon particular stimuli. Although the mechanisms underlying latency establishment and maintenance are very different for members of the different herpesvirus subfamilies, herpesvirus latency invariably is associated with a restricted viral gene and protein expression program that does not lead to infectious virus production. Given the importance of the m^6^A epitranscriptome in post-transcriptional regulation of protein production, it is plausible that differences in m^6^A methylation of transcripts may contribute to the switch in viral protein production program in latent versus lytic infection for at least some of these viruses. So far, this has only been extensively investigated in different latency/reactivation models of the gammaherpesviruses EBV and KSHV, possibly because, in contrast to other herpesviruses, latency is often the default pathway in cell culture for gammaherpesviruses and gammaherpesviruses have a robust latent transcriptome (94). Nonetheless, it may be interesting in future research to assess whether the m^6^A epitranscriptome may or may not contribute to latency/reactivation cycles in members of the different herpesvirus subfamilies, *in vitro* and particularly *in vivo*.

Interestingly, the current evidence indicates some marked similarities as well as some substantial differences in the way how different herpesvirus subfamily members interact with the m^6^A epitranscriptome. Specifically, alphaherpesviruses like HSV-1, PRV, and MDV appear to stand out as these viruses, unlike beta- or gammaherpesviruses, seem to avoid m^6^A methylation of (a substantial fraction of) their own transcripts and even disrupt the m^6^A methylation complex late in infection (38–41). Although the mechanisms underlying this inhibition of m^6^A methylation have to some extent been resolved (38–40), its consequences for virus biology and pathogenesis remain largely unexplored. Since, among herpesviruses, alphaherpesviruses also stand out by triggering a preferential degradation of m^6^A-methylated host transcripts (76), a speculative explanation for these observations may be that alphaherpesviruses trigger degradation of m^6^A-methylated host transcripts to suppress the antiviral IFN/ISG response, while their inhibition of the m^6^A methylation machinery and the reduced occurrence of DRACH m^6^A consensus sequences in their genomes may serve as safeguard mechanisms to avoid excessive degradation of viral transcripts.

Unlike PRV, HSV-1 or MDV, the betaherpesvirus HCMV does not inhibit m^6^A methylation. HCMV infection is also not associated with downregulation of m6A-associated proteins, such as YTHDF2, which has been linked with the fact that HCMV lacks host shutoff activity, unlike other viruses like HSV-1 and vaccinia virus (51). Quite contrary, and possibly because of this lack of host shutoff, HCMV infection leads to increased expression of m^6^A writer complex components and m^6^A readers, although m^6^A methylation of viral transcripts does not appear to play a critical role in virus replication (47). Current evidence supports a model where HCMV infection, either actively or due to a lack of host shutoff, is associated with an upregulation of host factors involved in m6A methylation which promotes cellular degradation of m^6^A-methylated IFN and ISG transcripts, thereby suppressing the antiviral host response (46, 47). Hence, although the underlying mechanisms are different and to some extent opposite, both alpha- and betaherpesviruses appear to manipulate or benefit from the m^6^A methylation machinery to suppress the antiviral IFN/ISG response.

For gammaherpesviruses, the role of m^6^A transcript methylation has been mainly studied in the context of latency/reactivation models of EBV and KSHV. This has revealed some intriguing apparent differences between both viruses. For KSHV, m^6^A methylation plays an important role in reactivation, since m^6^A methylation is important for correct splicing and expression of the major viral transactivator RTA, and RTA expression in turn promotes m^6^A methylation of viral lytic transcripts, thereby contributing to lytic viral replication (49). For EBV, on the other hand, m^6^A methylation of (lytic) viral transcripts is mainly associated with their degradation, thereby predominantly promoting viral latency (61). At the same time, m^6^A methylation also promotes production of particular viral proteins that are involved in EBV latency, such as EBNA2 (63). Both KSHV and EBV infection are associated with an overall reduction in m^6^A methylation of host transcripts. However, in both cases, infection also leads to increased m^6^A methylation of a selection of host transcripts. The mechanisms underlying this apparent finetuned and selective modulation of m^6^A methylation of host transcripts are currently not understood, and their investigation may reveal crucial information regarding selectivity of mRNA m^6^A methylation in general. In any case, several of the host transcripts that show either increased or reduced m^6^A methylation during KSHV or EBV infection are related to the antiviral host response, and the change in their m^6^A methylation profile in infected cells contributes to suppression of the host response (63, 84, 86, 87, 89). Consequently, manipulation of the m^6^A epitranscriptome to suppress the antiviral IFN/ISG response emerges as a universal theme that holds true for members of all three herpesvirus subfamilies.

Although the current review focuses on the interaction of herpesviruses with m^6^A methylation of mRNA, different reports indicate that herpesviruses may also make use of and affect m^6^A methylation of noncoding RNA molecules, including long noncoding RNAs (lncRNAs), short nuclear RNAs (snRNAs), and microRNAs. For example, in HCMV-infected Kasumi-3 lymphoblast cells, binding of YTHDF2 to m^6^A-methylated viral lncRNAs increases lncRNA stability, which has a proviral function (95); infection of chicken embryo fibroblasts with MDV increases m^6^A methylation of long noncoding host RNAs (96); and infection of porcine epithelial cells with PRV results in strongly reduced m6A levels in host snRNA (80). Also, for EBV, m^6^A methylation of the viral microRNA BART3-3p stimulates proliferation of NKTCL cells and may therefore promote EBV-associated NK/T cell lymphoma growth (97).

Due to the millions of years of coevolution with their hosts, herpesviruses can be considered “excellent cell biologists.” Tracing down the complex interactions of these viruses with particular host cell processes, such as m^6^A methylation of transcripts, may therefore reveal novel and important insights in the functions and regulation of these cellular processes. For example, HCMV, HSV-1, and PRV studies have been critical in revealing and confirming the important regulatory role of the m^6^A epitranscriptome in antiviral IFN/ISG responses (47, 73, 76, 80). Also, studies on alphaherpesviruses have revealed that phosphorylation may play a key role in regulating the activity of m^6^A writer and eraser proteins (38, 41, 80). Such information may have consequences that stretch beyond virology as they may be relevant in many other fields that are affected by the m^6^A epitranscriptome, including inflammatory diseases (98), cancer (99), and diabetes (100).
